# Milling Parameters and Quality of Machined Surface of Wire Arc Additive Manufactured AISI 321 Steel

**DOI:** 10.3390/ma19030567

**Published:** 2026-02-02

**Authors:** Qingrong Zhang, Victor Nikolaevich Kozlov, Vasiliy Aleksandrovich Klimenov, Dmitry Anatolyevich Chinakhov, Roman Vladimirovich Chernukhin, Zeli Han, Mengxu Qi

**Affiliations:** 1 Division for Materials Science, School of Advanced Manufacturing Technologies, National Research Tomsk Polytechnic University, Tomsk 634050, Russia; cinzhun1@tpu.ru (Q.Z.); hanzelizy@gmail.com (Z.H.); 2 Department of Mechanical Engineering, School of Advanced Manufacturing Technologies, National Research Tomsk Polytechnic University, Tomsk 634050, Russia; kozlov-viktor@bk.ru (V.N.K.); mensyuy1@tpu.ru (M.Q.); 3 Faculty of Aircraft Engineering, Novosibirsk State Technical University, Novosibirsk 630073, Russia; 4 The Faculty of Mechanical Engineering and Technologies, Novosibirsk State Technical University, Novosibirsk 630073, Russia; chernuxin@corp.nstu.ru

**Keywords:** wire arc additive manufacturing, austenitic stainless steel, milling, machining parameters, cutting forces, surface roughness, temperature, vibration

## Abstract

Due to the unique microstructure and mechanical heterogeneity of austenitic stainless steel made via wire arc additive manufacturing (WAAM), its machinability differs significantly from that of rolled material. Accordingly, this study systematically investigates the influence of milling strategies on key process responses (cutting forces, surface roughness, vibration displacement, and temperature) to reveal the mechanisms of machining parameters during the milling of WAAM-fabricated austenitic stainless steel. The material used in this study is ER321 austenitic stainless steel. During deposition, the fusion zone cools more slowly than the transition zone; consequently, the fusion zone exhibits a hardness approximately 20 HV_0.1_ lower than that of the transition zone. Surface roughness is primarily reduced by decreasing the primary feed per tooth. However, when the primary feed per tooth is small, ploughing is induced, which not only increases surface roughness by 25% but also causes abnormal increases in temperature and vibration displacement. Nevertheless, ploughing has little effect on the total milling force, and the feed per tooth shows a positive correlation with the total milling force. Tool run-out and an increase in the uncut chip thickness lead to a positive correlation between the radial depth of cut and the key process responses. Moreover, ploughing also occurs when the radial depth of cut is small. The axial depth of cut has almost no effect on the machining process. Moreover, a small-diameter mill leads to severe ploughing, and at a high table feed, climb milling leads to cutter offset.

## 1. Introduction

With the growing industrial demand for lightweight parts with a complex structure, additive manufacturing is being widely applied in mechanical engineering, aerospace, and biomedicine, due to its high design freedom, short production cycles, and low material waste. Wire arc additive manufacturing (WAAM), a wire-based directed energy deposition technique, has become one of the most promising metal AM processes. In WAAM, a continuous metal wire is melted by an electric arc and deposited layer by layer onto a substrate to build fully dense three-dimensional parts. The deposition path is precisely controlled via a pre-programmed robotic arm. Since WAAM is based on conventional welding technology, it uses standard welding consumables and off-the-shelf power sources, resulting in significantly lower costs, typically an order of magnitude less than those of selective laser melting (SLM). Moreover, the higher thermal input of the electric arc compared to laser enables exceptionally high deposition rates, reaching up to 800 g/min. With the use of robotic arms, part size is no longer limited by the printer’s build chamber [1,2].

During the WAAM process, the concentrated heat input, steep thermal gradients, and repeated thermal cycles lead to the formation of the microstructure and mechanical properties significantly differing from those of rolled materials, which are usually characterized by coarse grains, heterogeneity, and anisotropy [3,4]. Deposited titanium alloys exhibit a strong <001> texture along the growth direction, with columnar β grains up to 4 mm wide and 10 mm high, extending across multiple layers [5]. The hardness difference between the edge and the center of the sample can reach 140 HV [6]. Despite the absence of columnar grains covering multiple layers, deposited austenite still has <100> texture with a significantly different microstructure in different zones. Thus, in the fusion zone, δ-ferrite primarily appears with a vermicular and lathy morphology, while at the fusion interface, δ-ferrite has a columnar and globular structure [4]. In different zones, the hardness reaches 30 HV [7]. In our recent research [8], grain refinement was performed through heat treatment after deposition, but we still failed to eliminate columnar β grains. In [9], the authors tried to modify the heat dissipation by adding side limiters (shaping blocks); however, microstructure heterogeneity remained, while the hardness difference reached 20 HV.

Furthermore, the surface quality of wire-arc-deposited parts is poor and has significant waviness, with the peak-to-valley height exceeding 1 mm [1]. Therefore, the surface must be machined to meet engineering requirements. However, due to the unique properties of the deposited material, its machinability significantly differs from that of a rolled material. The deposited Ti6Al4V alloy possesses a lower work hardening owing to its porosity [10]. Due to high cooling rates used in the deposition process, AISI H13 undergoes martensitic transformation, resulting in a 180% higher hardness and 24% greater cutting force compared to a rolled material [11]. High-strength, low-alloy steel deposited with AWS-A5.28 ER110S-G welding wire possesses higher ductility, leading to more significant plastic flow during machining, which, in turn, results in pronounced burrs and higher adhesion [12]. In addition to the material properties, the surface geometry of the deposited sample is another important factor influencing the machining process. The surface waviness leads to periodic fluctuations in cutting forces, in contrast to the stable cutting forces observed during the machining of flat surfaces [13].

Austenitic stainless steel is one of the materials most widely used in additive manufacturing due to its high strength, excellent corrosion resistance, and good weldability. It finds extensive applications in the automotive industry, biomedicine, and nuclear engineering, producing components such as automotive cross members, coronary stents, and shielding modules for nuclear reactor systems [14]. However, austenitic stainless steel exhibits poor thermal conductivity and high ductility, which lead to high cutting forces, difficulty in chip formation, severe tool wear, and a tendency for built-up edge adhesion during machining [15]. Meanwhile, the machinability of AM-produced (especially deposited) austenitic stainless steel has been insufficiently studied, and many researchers focus on process parameters and properties of the material. Little attention has been paid to machining parameters such as feed rate and spindle revolutions per minute (rpm). Dabwan et al. [16] compare the machinability of 316L stainless steel synthesized by selective laser melting (SLM) in different machining directions. They show that the cutting force, surface roughness, and hardness differ by 51, 34, and 14.7%, respectively. Laue et al. [17] compare the machinability of 316L stainless steel fabricated via different methods (WAAM, SLM, rolling). They find that the cutting force is the highest when milling rolled steel, while its surface quality is high. Diaz-Plaza De Los Reyes et al. [18] investigate the influence of input energy volumetric density on cutting forces. They show that a 6% increase in the energy density results in a 1% increase in the cutting force. Gong et al. [19] investigate the tool wear when milling 316L stainless steel produced by laser additive manufacturing. They point out that pores and cracks in the structure lead to the tool chipping and crushing.

Although Struzikiewicz et al. [20] studied the influence of several machining parameters on the machining of printed stainless steel using the Taguchi method, turning was the specific machining method of limited applicability. In contrast, Qi et al. [21] investigated the influence of machining parameters on the milling force during the milling of printed stainless steel; however, they did not explore the surface quality of martensitic stainless steel characterized by relatively poor ductility.

Using more widely applicable milling [21], the present study systematically and thoroughly examines the effect of the main machining parameters (feed rate, spindle rpm, and radial and axial cutting depths) on the machining of difficult-to-process AISI 321 austenitic stainless steel [15]. This steel contains titanium (Ti), which suppresses chromium carbide precipitation during thermal exposure, thereby mitigating intergranular corrosion and enabling its use in the manufacture of high-temperature components such as turbocharger housings and heat exchangers [22]. In this work, the steel hardness is determined by average values in different zones, which are selected in accordance with the properties of the deposited austenitic stainless steel, rather than according to the overall average [16,17,20]. It is shown that cutting forces, temperature, and vibration interrelate and mutually influence surface roughness. These findings are further confirmed by the machined surface and chip morphology and changes in the momentary cutting forces, depending on the angular position of the tool teeth.

## 2. Materials and Methods

### 2.1. Fabrication of Samples

**Figure 1:**
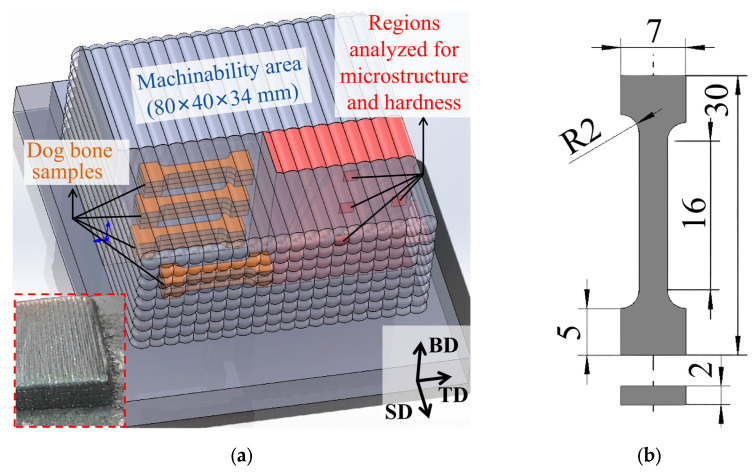
Areas designated for various testing and the cutting of dog-bone samples (**a**) and a dog-bone sample used for tensile strength testing (**b**). BD—build direction, SD—scanning direction, TD—transverse direction. Inset: a photograph of the as-deposited specimen.

The deposition process was performed using a cold metal transfer (CMT) welding power source [Fronius TPS 400i (Fronius, Pettenbach, Austria)] with a KUKA R1810 (KUKA, Augsburg, Germany) robotic manipulator. A low-carbon steel Q235B (10 mm thick) was used as a substrate to reduce experimental costs, as its influence on the microstructure and properties of the deposited area more than 4 mm above its surface was negligible according to the results reported in [14]. ER321 austenitic stainless-steel wire with a diameter of 1.2 mm was used for deposition. The chemical composition of both materials is given in Table 1. The deposited sample is shown in Figure 1a, with dimensions of 80 mm × 80 mm × 34 mm. The deposition path and parameters were obtained from [21,23] and adjusted using the sample dimensions and the wire diameter by modifying the welding speed. The process parameters are presented in Table 2. The sample was not heat-treated after deposition.

### 2.2. Characterization of Microstructure and Mechanical Properties

Prior to the experiment, the microstructure and mechanical properties of the steel sample were characterized to avoid its heterogeneity effect [4] on milling results. Dog-bone tensile samples, as well as the areas selected for microstructural and hardness analyses, were all extracted from the top, middle, bottom, and edge regions of the deposited sample. Tensile tests were performed along the TD. Both microstructural observations and microhardness measurements were carried out on the BD–PD plane. The microstructure was studied using an Axio Observer A1m optical microscope (Carl Zeiss, Oberkochen, Germany) following etching with aqua regia (67 vol.% HNO_3_ and 33 vol.% HCl). Tensile strength was measured using an MIM.4 universal testing machine (MIM, St Petersburg, Russia) at a crosshead speed of 2 mm/min. Three dog-bone tensile samples were tested in each region. Their dimensions are shown in Figure 1b. The sample hardness was measured using an EMCO-TEST Durascan-10 microhardness tester (EMCO-TES, Kuchl, Austria) at 0.1 kgf load and 10 s exposure. Three measurements were performed at different zones within each region.

### 2.3. Machinability Testing

Before machinability testing, the sample was pre-milled to gain a flat reference surface. Pre-milling was conducted on a CONCEPT Mill 155 CNC milling machine (EMCO, Hallein, Austria) using four-flute cemented carbide end mills (90 wt.% WC, 10 wt.% Co) with diameters of 12 mm and 8 mm. End mills were coated with an AlTiN layer deposited via physical vapor deposition (PVD). The helix, rake angle, clearance angle, and overhang length of the end mills were 40 degrees, 10 degrees, 16 degrees, and 35 mm, respectively. Machinability testing was also conducted using the end mills on the same CNC milling machine as mentioned above.

**Figure 2:**
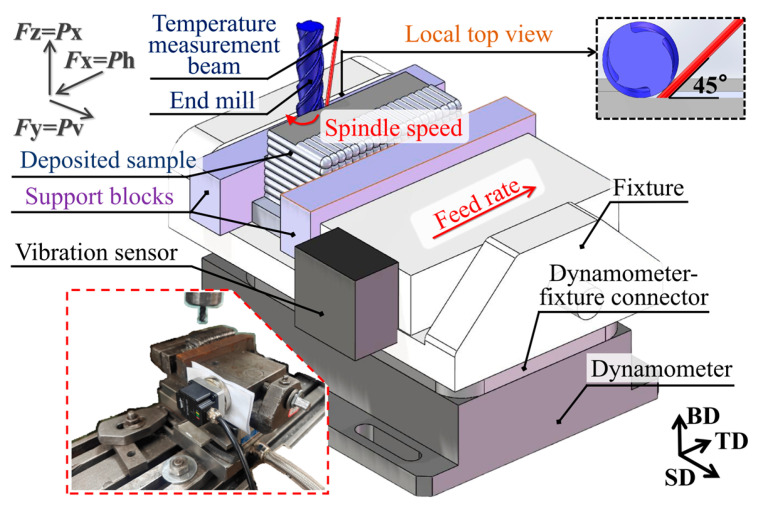
Measurement setup for the milling process. Inset: a photograph of the setup.

Figure 2 illustrates the measurement setup. Cutting forces were measured using a Kistler 9257B dynamometer (Kistler, Winterthur, Switzerland) with a sensitivity of 7.5 N and measurement error of ±0.005%. The dynamometer outputs, forces *F*x, *F*y, and *F*z, correspond to the feed force *P*h in the feed direction (TD); the transverse force *P*v is perpendicular to the feed (SD) direction; and the axial force *P*x is in the direction of the end mill axis (BD direction). The table feed *v*_f_ (mm/min) and the primary cutting feed per tooth *f*_zae_ (mm/tooth) are in the same direction. They are theoretically proportional, as shown in Equation (1):

(1) $$f_{zae}=v_{f}\times \frac{arc(1-2\times a_{e}/d)}{360\times n},$$

where *a_e_* (mm) is the radial cutting depth, *d* (mm) is the diameter of the end mills, and *n* (rpm) is the spindle speed [24].

Temperature was measured with a single-point infrared thermometer YCR-D2080AR (Wuxi Youtian Environmental Technology, Wuxi, China) with an emittance of 0.39 [25]. The infrared beam was oriented at a 45-degree angle to the machined surface and tangential to the end mill (Figure 2). The measurement point was located at the center height of the machined surface. The sample vibration displacement was measured using a WT-VB02-485 vibration sensor (WitMotion, Shenzhen, China). Since the feed motion was driven by the worktable (rather than the milling cutter spindle), the sample was more susceptible to vibration than the end mill, so the sensor was mounted on the fixture, rather than on the milling cutter spindle. The surface roughness *Ra* at the center height of the machined surface was measured with a TR200 tester (JITAI, Shanghai, China), using a Gaussian filter with an evaluation length of 4 mm and a cutoff length of 0.8 mm. The obtained value was averaged by three measurements. The machined surface topography and chip morphology of the sample were observed using a GP-304K microscope (KSGAOPIN, Kunshan, China).

Five repeated tests were conducted under the same mode, each lasting over 10 s [20]. The final result for each mode was taken as an average value obtained from each of the five tests. Given that the relative standard deviation of the five tests was consistently below 5%, indicating good data repeatability, the confidence interval is not shown in the line plots presented in the sections below.

Given the many machining parameters involved, leading to high experimental costs and reduced efficiency with the general orthogonal designs employed [20], a control-variable approach was used in the experiment, wherein only one parameter was varied, while all other parameters were specified. The selection of parameter values was guided by [21], and the detailed machining plan is given in Table 3.

When designing the plan for evaluating the table feed’s influence on the milling process, the spindle rpm was appropriately increased to reduce the primary cutting feed per tooth, thus lowering the risk of edge chipping under large axial cutting depths *a_p_* [26]. When designing the plan for evaluating the cutting configuration’s influence on the milling process, the cutter diameter was reduced to enhance the sensitivity of the vibration displacement to the milling strategy [27].

## 3. Results and Discussion

### 3.1. Microstructure and Mechanical Properties of the Deposited Sample

**Figure 3:**
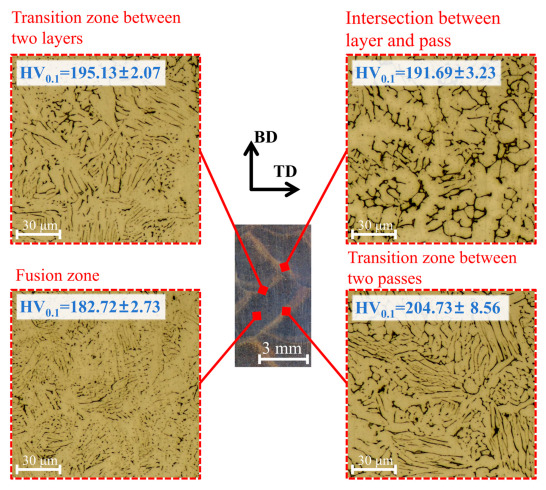
Optical images of morphology at the sample center.

According to optical images, the difference in the microstructure at the sample center, at the sample edge, and at different heights is insignificant. Therefore, Figure 3 presents the morphology only for the central region, where δ-ferrite is granular in the fusion zone, lathy near the transition zone (δ-ferrite near the transition zone between two passes is coarser than that near the transition zone between two layers), and vermicular at the intersection between layer and pass. Moreover, compared with the transition zone, δ-ferrite in the fusion zone exhibits a finer morphology and a denser distribution, whereas δ-ferrite at the layer–pass intersection exhibits a coarser morphology and a sparser distribution.

References [7,28] provide an explanation for these morphological differences in δ-ferrite. In the transition zone, which is near the weld bead surface and exposed to the shielding gas during deposition, the cooling rate is high. This causes solidification to proceed via the ferrite–austenite mode (FA): primary δ-ferrite forms first from the melt, followed by a eutectic transformation that simultaneously precipitates both ferrite and austenite, resulting in the formation of lath δ-ferrite. As the solidification front advances toward the interior of the weld bead, the cooling rate decreases and solidification shifts to the austenite–ferrite mode (AF). In this mode, primary austenite forms first, followed by a eutectic transformation that simultaneously precipitates both ferrite and austenite. This leads to the formation of granular δ-ferrite.

Due to the layer-by-layer deposition process, the interlayer cooling time is longer than the interpass cooling time, resulting in a significantly faster rise in the valley temperature of the thermal cycle in the transition zone between two passes compared to that between two layers [29,30]. Consequently, in the transition zones solidified via the FA mode, the δ-ferrite laths in the zone between two passes are thicker than those in the zone between two layers, leading to higher hardness. The intersection between layer and pass undergoes remelting twice—once during the deposition of the adjacent weld bead, and once during the deposition of the overlying weld bead. This double remelting leads to coarser δ-ferrite and grain growth, thereby resulting in reduced hardness. Furthermore, reference [7] notes that the fusion zone exhibits significant residual tensile stresses, which lead to its lower hardness.

In addition, strength and hardness values in different zones of the sample show no significant differences (relative standard deviation < 1.5%). At the center, the strength is 621.66 ± 3.06 MPa.

### 3.2. Influence of the Table Feed During the Milling Process

**Figure 4:**
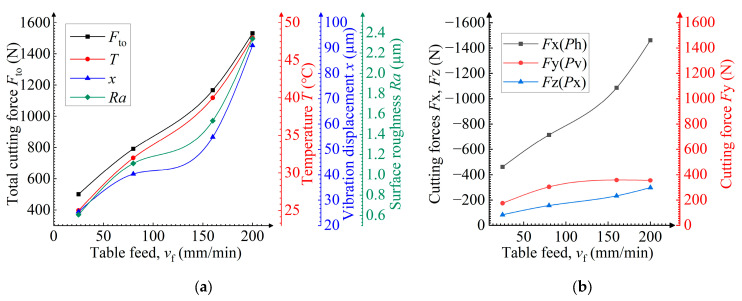

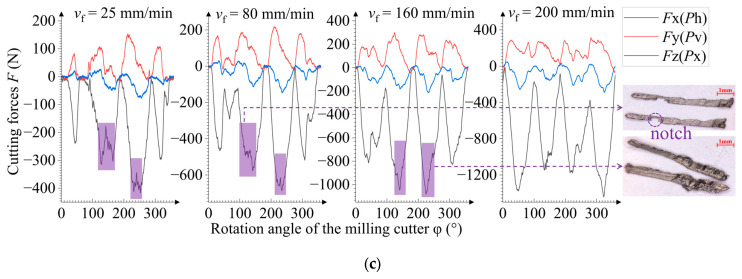
Dependences of the table feed *v*_f_ on (**a**) the total cutting force *F*_to_, temperature *T*, vibration displacement *x*, and surface roughness *Ra*, and on (**b**) cutting forces *F*x, *F*y, *F*z; (**c**) dependences of the momentary cutting force with the rotation angle φ of the cutter. The purple color indicates cutting force fluctuation. Inset: chips.

**Figure 5:**
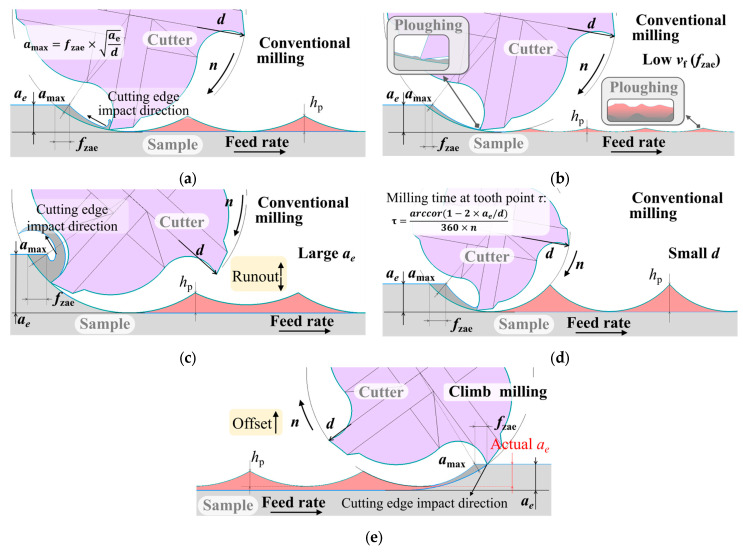
Maximum uncut chip thickness *a*_max_ and peak height *h*_p_ with different table feeds *v*_f_ (primary feed per tooth *f*_zae_) (**a**,**b**), radial cutting depths *a*_e_ (**c**), milling cutter diameters *d* (**d**), and milling strategies (**e**).

Dependences between the main parameters and the table feed *v*_f_ are shown in Figure 4a. The total values of the cutting force and vibration displacement are the highest selected from the vector summation of the measurement results in orthogonal directions using a square-root method. The main parameters tend to grow as the table feed increases. This dependence is explained by Malkin and Guo [24]: they report that the increased values of total cutting force and temperature result from the higher maximum thickness *a*_max_ of uncut chips. The higher vibration displacement is determined by the growth in cutting force, and the higher surface roughness results from an increase in the height *h*_p_ of the peaks not removed by the cutter (Figure 5a,b). At *v*_f_ = 160 mm/min, the growth in the main parameter values slows due to the dying-away of momentary cutting force fluctuations during tooth engagement. The dying-away of fluctuation correlates with the disappearance of incomplete cutting. The presence or absence of notches in the chip can identify an approach cut (Figure 4c). The dying-away of fluctuation is not observed during the milling of the rolled sample, which can be attributed to the more uniform distribution of strength and hardness in the rolled material [16,31]. In Figure 4c, tooth engagement and disengagement relate to the overall rise and fall in the momentary cutting force, respectively [21]. The difference in the maximum cutting force for each tooth is caused by tool run-out or by misalignment in its installation in the collet chuck [31].

Figure 4b shows the direction (represented by +/−) of the cutting force components and their dependence on the table feed *v*_f_. The presented values of the cutting force components (excluding +/−) are the highest values selected from the absolute values of final measurements. Negative feed and axial forces and the positive transverse force indicate that the main impact of the cutting edge on the sample is opposite to the feed direction, axially upward and laterally outward (Figure 2). This is a typical direction for the cutting-edge’s impact during conventional milling with a low radial cutting depth [31]. With increasing table feeds, the transverse force *P*v and axial force *P*x are low and remain almost constant, whereas the feed force *P*h is high and significantly grows. This is because it is most involved in removing the metal allowance, while the cutting edge tends to act against the feed direction in this milling mode (Figure 5a).

### 3.3. Influence of the Spindle rpm During the Milling Process

**Figure 6:**
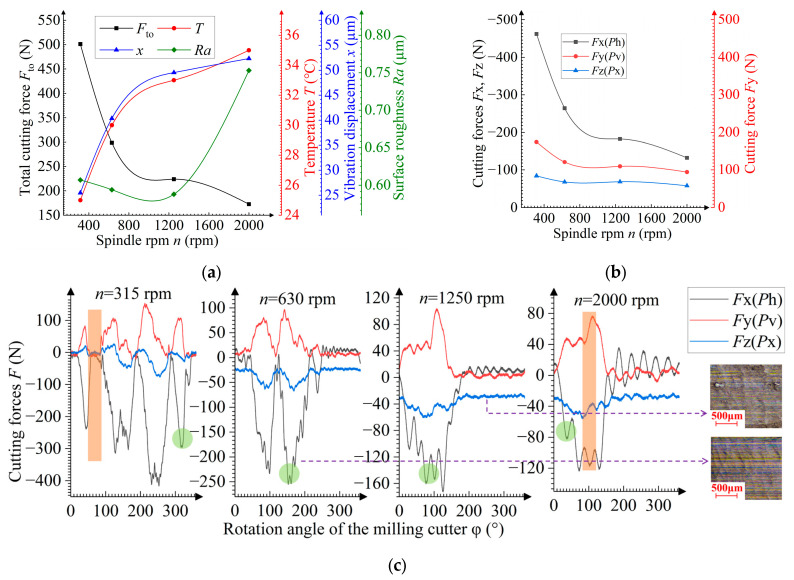
Dependences of the spindle rpm *n* on (**a**) the total cutting force *F*_to_, temperature *T*, vibration displacement *x*, and surface roughness *Ra*, and on (**b**) cutting forces *F*x, *F*y, *F*z; (**c**) dependences of the momentary cutting force *F* on the rotation angle φ of the cutter. Green circles indicate cutting force peaks, and the brown color indicates the exit of one tooth and entry of the next one. Inset: machined surface.

At *n* < 1000 rpm, the cutting force decreases with increasing *n* due to a reduction in the uncut chip thickness *a*_max_ caused by a decrease in the primary tooth feed *f*_zae_. The surface roughness lowers due to the reduced peak height *h*_p_ (Equation (1) and Figure 5b). At spindle rpm *n* values ranging between 1000 and 1400 rpm, the surface roughness tends to grow due to more intensive ploughing, and the descent rate of the cutting force approaches zero. The ploughing phenomenon occurs when milling with a low primary cutting feed per tooth *f*_zae_, when chips are not formed properly, and the material undergoes plastic strain and indents under the cutting edge, thereby increasing the surface roughness [32]. The higher ploughing intensity is shown in Figure 6c. The number of high peaks of the cutting force reduces as the spindle rpm increases. This translates to an increase in the teeth number subjected to chip-free cutting, and thus an increased amount of the material pressed under the cutting edge and in the cutting surface, which then transfers to the machined surface. According to Figure 6c, the machined surface has ploughing traces. Within the theoretical angular interval between the exit of one tooth and the entry of the next one, the overall fall and rise in the cutting force (at *n* = 315 rpm) is absent at *n* ≥ 1250 rpm. When *n* > 1400 rpm, the cutting force decreases due to a further reduction in *f*_zae_. This, however, does not mitigate the ploughing intensity, and the surface roughness continues to grow. The high ploughing intensity results in a 25% increase in the surface roughness.

At *n* < 800 rpm, the vibration displacement and temperature increase due to the higher linear velocity *v* of the cutting edge [24,33]. Furthermore, reference [34] indicates that an increase in the cutting speed also leads to a higher degree of austenite-to-martensite transformation. The dependence between the velocity *v* (mm/min) and spindle rpm *n* is calculated from [24] as follows:

(2) v=π·d·n,

where *d* is the diameter (mm) of the milling cutter.

At *n* > 800 rpm, the vibration displacement slows down due to the reduced cutting force. The temperature rise also slows down due to the enhanced heat dissipation caused by the reduced primary cutting feed per tooth.

### 3.4. Influence of the Radial Cutting Depth During the Milling Process

When the radial cutting depth *a*_e_ grows, the higher uncut chip thickness *a*_max_ leads to an increase in the cutting force and temperature (Figure 5c). The radial depth growth also leads to lower heat removal, resulting in a further temperature rise.

**Figure 7:**
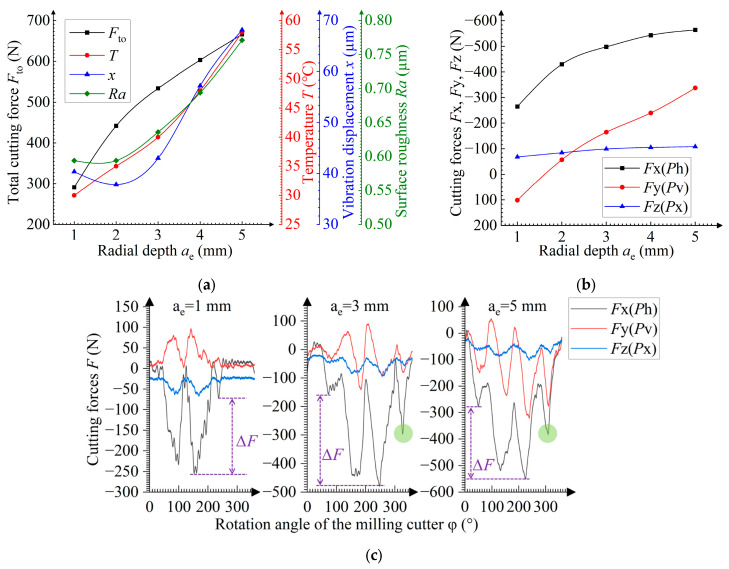
Dependences of the radial depth *a*_e_ on (**a**) the total cutting force *F*_to_, temperature *T*, vibration displacement *x*, and surface roughness *Ra*, and on (**b**) cutting forces *F*x, *F*y, *F*z; (**c**) dependences of the momentary cutting force *F* on the rotation angle φ of the cutter. Green circles indicate cutting force peaks.

The ploughing elimination at *a*_e_ < 2 mm reduces vibration displacement and hinders any increase in the surface roughness. The ploughing elimination is evidenced by the increasing number of cutting force peaks in Figure 7c. At *a*_e_ > 2 mm, the cutting force growth leads to higher vibration, while an increase in the tool run-out enhances the surface roughness (Figure 5c). This increase in the tool run-out is confirmed by the growing difference in cutting force peaks Δ*F*, as shown in Figure 7c.

Moreover, the transverse force transition from positive to negative with increasing radial depth *a*_e_ indicates that the transverse action of the cutting edge shifts from pushing outward to pulling inward (Figure 2). The feed force slowly increases and the significantly growing transverse force suggests that the cutting edge’s impact on the sample changes from primarily opposing the feed direction to a primarily transverse direction as the exit angle of the cutting edge increases (Figure 5c).

### 3.5. Influence of the Axial Cutting Depth During the Milling Process

**Figure 8:**
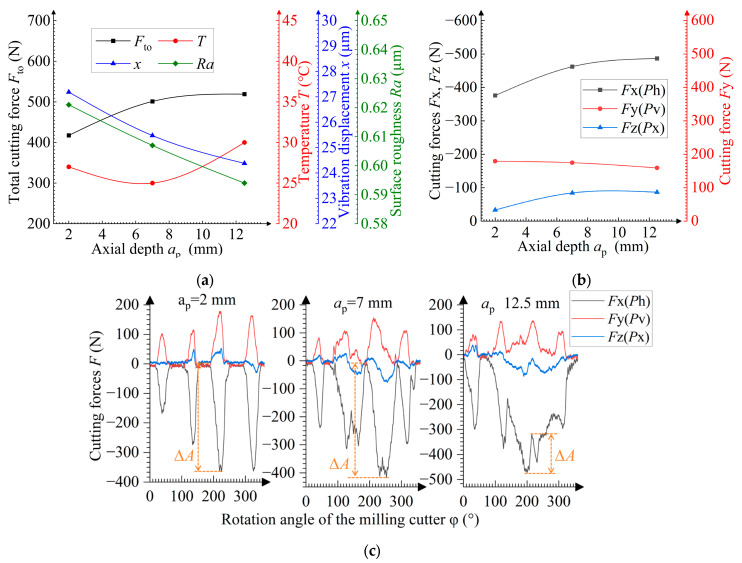
Dependences of the axial depth *a*_p_ on (**a**) the total cutting force *F*_to_, temperature *T*, vibration displacement *x*, and surface roughness *Ra*, and on (**b**) cutting forces *F*x, *F*y, *F*z; (**c**) dependences of the momentary cutting force *F* on the rotation angle φ of the cutter.

**Figure 9:**
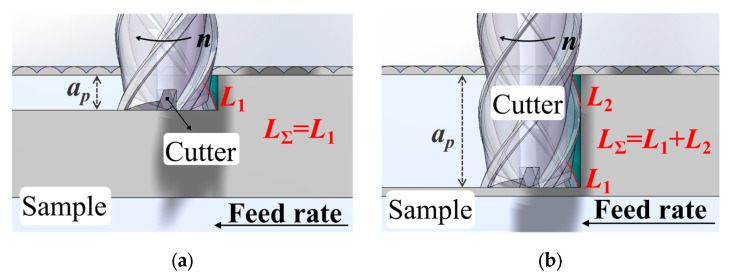
Total length *L*_Σ_ of the cutting edge when milling at small (**a**) and large (**b**) axial depths *a*_p_.

The influence of the axial cutting depth *a*_p_ on the machining process is shown in Figure 8. The growth in the total length *L*_Σ_ involved in milling is observed for the total cutting edge with increasing *a*_p_, leading to a higher cutting force (Figure 9). The *L*_Σ_ growth indicates a higher contact length between the sample and the cutter, which reduces the milling irregularity and suppresses the vibration, thereby decreasing the surface roughness [35]. This vibration suppression can also be indicated by the reduced cutting force amplitude Δ*A* (Figure 8c). Additionally, at *a*_p_ ≤ 7 mm, the vibration reduction provides a temperature decrease [36]. However, at *a*_p_ ≥ 7 mm, the increased contact length *L*_Σ_ leads to an increase in the heat generation, while changes in the balance between the heat generation and dissipation cause the temperature to rise. In comparison with the milling of high-hardness materials such as martensitic stainless steel, the effect of the axial cutting depth on milling is less pronounced [21].

### 3.6. Influence of the Cutter Diameter During the Milling Process

**Figure 10:**
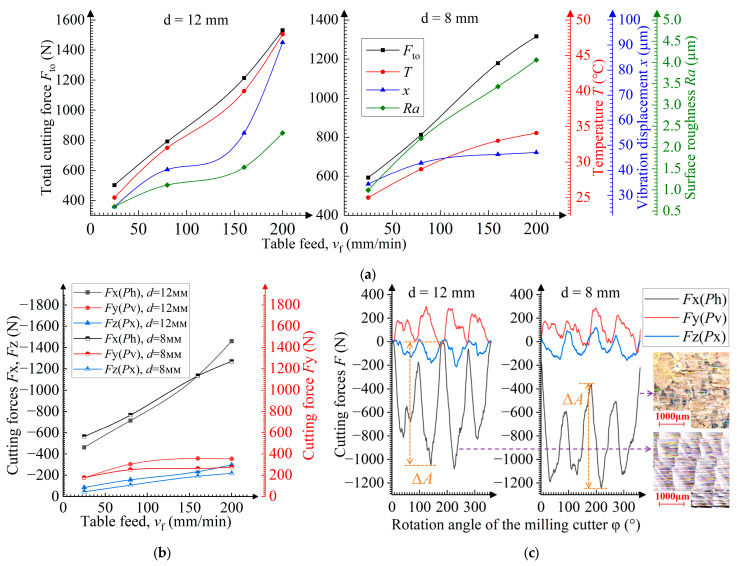
Dependences of the cutter diameter *d* on (**a**) the total cutting force *F*_to_, temperature *T*, vibration displacement *x*, and surface roughness *Ra*, and on (**b**) cutting forces *F*x, *F*y, *F*z; (**c**) dependences of the momentary cutting force *F* on the rotation angle φ of the cutter at *v*_f_ = 160 mm/min. Inset: machined surface.

When using a small-diameter cutter, the peak height *h*_p_ is higher due to the small curvature radius that results in higher surface roughness. These peaks are steeper and thus more easily pressed in the transient surface (ploughing), thereby further increasing the surface roughness (Figure 5d and Figure 10c). The difference in the surface roughness reaches 50% compared to that obtained with a large-diameter cutter. However, the smaller diameter leads to better heat dissipation, which reduces the temperature during milling [37].

According to the equation in Figure 5d, the smaller the cutter diameter, the longer the milling time τ at the tooth point and, hence, the longer the simultaneous multi-tooth engagement time, which reduces the cutting force amplitude Δ*A* (Figure 10c) and vibration displacement. However, according to the equation in Figure 5a, the smaller the cutter diameter, the higher the uncut chip thickness *a*_max_, which results in a larger cutting force. Under the combined effect of these two factors, the vibration displacement is greater when the table feed *v*_f_ ≤ 100 mm/min, but the vibration displacement is smaller when *v*_f_ > 100 mm/min. This smaller vibration displacement at *d* = 8 mm also results in the lower cutting force observed at *v*_f_ > 150 mm/min (Figure 10a).

### 3.7. Influence of the Strategy During the Milling Process

**Figure 11:**
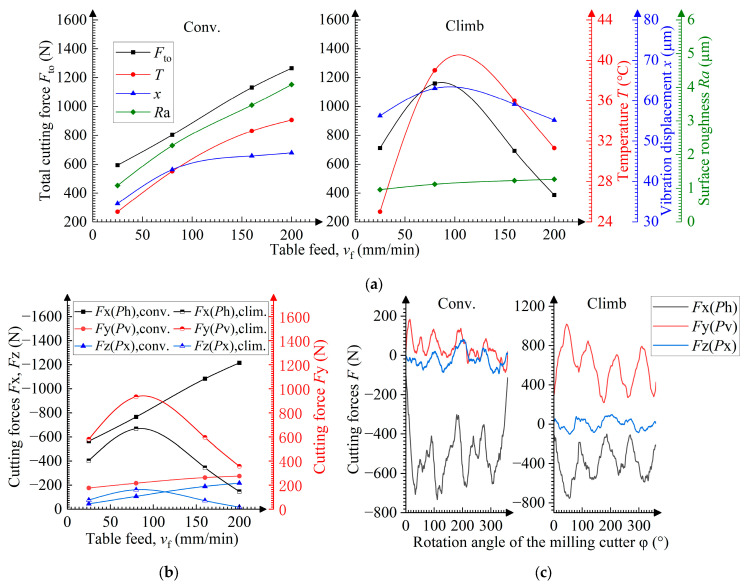
Dependences of the milling strategy on (**a**) the total cutting force *F*_to_, temperature *T*, vibration displacement *x*, and surface roughness *Ra*, and on (**b**) cutting forces *F*x, *F*y, *F*z; (**c**) dependences of the momentary cutting force *F* on the rotation angle φ of the cutter at *v*_f_ = 80 mm/min.

**Figure 12:**
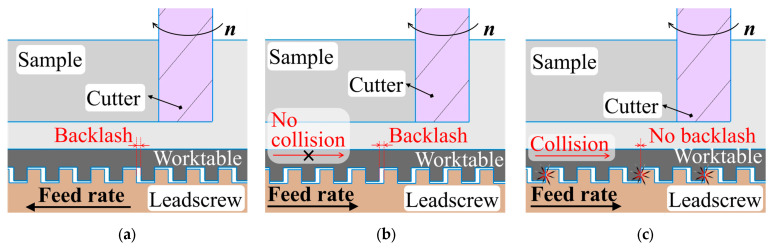
Backlash position in (**a**) conventional milling and (**b**) climb milling; (**c**) the moment of the cutter tool engagement during climb milling.

Compared to conventional milling, climb milling results in lower surface roughness due to the absence of ploughing (Figure 11a), but it induces higher vibration displacement due to collision between the lateral surfaces of the leadscrew thread and worktable nut thread, induced by backlash in the feed drive system (Figure 12). The difference in the surface roughness and the vibration displacement reaches 300 and 30%, respectively.

At *v*_f_ > 80 mm/min, both the feed and transverse forces decrease (Figure 11b), which is similar to the trend observed in the values of cutting force components as the radial cutting depth *a*_e_ decreases (Figure 7b,c). This indicates that the decrease in the cutting force is caused by a reduction in the actual radial cutting depth, resulting from the offset of a less rigid milling cutter under high cutting forces (Figure 5e).

The actual radial cutting depth reduction also leads to a slight decrease in the vibration displacement and causes the temperature to fall.

As shown in Figure 11b, the transverse force predominates in climb milling. This is because the cutting edge impacts the sample toward the feed lateral direction in the moment of engagement (Figure 5e).

### 3.8. Parameters of the Milling Process

The influence of different machining parameters on the milling of WAAM-fabricated austenitic stainless steel AISI 321 is summarized in Table 4. Since the table feed *v*_f_ directly affects the primary cutting feed per tooth *f*_zae_, its influence on the cutting force *F*_to_, surface roughness *Ra*, vibration displacement *x*, and temperature *T* is very high. The spindle rpm *n* not only directly affects *f*_zae_, but also determines the linear velocity *v* of the cutting edge. Consequently, it affects not only *F*_to_ and *Ra* but also *T* and *x* parameters. At a high spindle rpm, the extremely small *f*_zae_ leads to ploughing, thereby increasing the surface roughness.

The radial cutting depth *a*_e_ significantly affects the temperature *T*, as a higher radial cutting depth value impedes heat removal. Moreover, *a*_e_ directly affects the uncut chip thickness *a*_max_, thereby considerably influencing *F*_to_, which, in turn, impacts *x*. Since *a*_e_ does not directly affect *f*_zae_, its influence on *Ra* is weak. Since the axial cutting depth *a*_p_ affects neither *f*_zae_ nor *a*_max_, and the higher cutting force resulting from the increased length *L*_Σ_ coincides with a reduction in the vibration displacement, its overall effect on the *F*_to_, *Ra, x*, and *T* parameters is weak.

The cutter diameter *d* primarily affects the milling system stiffness, thereby significantly influencing the vibration displacement *x*. Moreover, differences in *d* lead to differences in the ploughing intensity, which, in turn, results in substantial differences in *Ra*. Since the milling strategy directly affects the ploughing intensity, it has a significant effect on the surface roughness *Ra*. When the table feed *v*_f_ is relatively high, the tool offset in climb milling leads to reductions in *F*_to_, *x*, and *T*, which are lower than those observed in conventional milling.

## 4. Conclusions

1. The microstructure and mechanical properties differ among different zones of the sample. Due to the slower cooling rate in the fusion zone compared to the transition zone, the solidification mode in the fusion zone is as follows: austenite precipitates first, followed by a eutectoid reaction that simultaneously forms both austenite and ferrite; in the transition zone, the solidification mode is as follows: ferrite precipitates first, followed by a eutectoid reaction that simultaneously forms both austenite and ferrite. Moreover, the fusion zone exhibits a microhardness approximately 20 HV_0.1_ higher than that of the transition zone. The microstructure and mechanical properties show little difference among different regions of the sample. The tensile strength is 621.66 ± 3.06 MPa.
2. The surface roughness *R*a of the machined surface is primarily influenced by the primary feed per tooth *f*_zae_ and shows a positive correlation with *f*_zae_. However, when *f*_zae_ is extremely small, ploughing occurs, leading to abnormally increased temperature values and vibration displacement. The surface roughness *R*a is less affected by radial depth *a*_e_ and axial depth *a*_p_. With increasing *a*_e_, *R*a slightly increases due to tool run-out. The use of a small-diameter milling cutter significantly intensifies ploughing, thereby severely deteriorating the machined surface and doubling the surface roughness. Compared with conventional milling, climb milling results in a smaller *R*a (up to 65% lower) due to less material being pressed into the rear flank of the cutter.
3. In conventional milling with a small radial depth *a*_e_, the feed force *P*h, primarily responsible for material removal, is relatively large and significantly influenced by machining parameters, whereas the transverse force *P*v and axial force *P*x are small and remain nearly constant. As *a*_e_ increases, the exit angle of the cutting edge increases substantially, causing a significant change in the transverse force *P*v, including a reversal in its direction and a notable increase in magnitude. Compared with conventional milling, climb milling also leads to a significant increase in *P*v due to the altered impact direction of the cutting edge on the sample. Moreover, this impact force is sufficiently large to cause cutter offset at high feed rates (*v*_f_ > 125 mm/min), which subsequently reduces temperature, vibration displacement, and cutting forces.
4. Both the feed and radial depth lead to an increase in the uncut chip thickness *a*_max_, which results in increases in the total force, temperature, and vibration displacement. When the axial depth *a*_p_ is increased, the uncut chip thickness *a*_max_ theoretically remains unchanged, and vibration displacement is suppressed due to the increased contact length between the cutting edge and the sample; therefore, the axial depth *a*_p_ shows a weak correlation with the cutting forces, surface roughness, and temperature.

## Figures and Tables

**Table 1:** Chemical composition of the substrate and welding wire.

	Element (wt.%)									
	C	Si	Mn	P	S	Cr	Ni	Mo	Cu	Ti
Welding wire	0.016	0.49	1.52	0.021	0.002	18.9	9.08	0.08	0.13	0.17
Substrate	0.18	0.16	0.45	0.019	0.019					

**Table 2:** Process parameters of WAAM + CMT deposition.

Process Parameter	Details	Value
Deposition power	Current	121 A
	Arc voltage	19.1 V
Speed	Welding speed	0.6 m/min
	Wire feed rate	4.5 m/min
Deposition path	Layer height	4 mm
	Pass width	4 mm
	Electrode-to-layer angle	90°
	Raster filling patterns	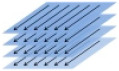
Shield gas	Shield gas	98% Ar, 2% CO_2_
	Flow rate	20 L/min

**Table 3:** Machining plan for the deposited sample.

Cutter Diameter, *d* (mm)	Milling Strategy	Table Feed, *v*_f_ (mm/min)	Spindle Rpm, *n* (rpm)	Radial Cutting Depth, *a*_e_ (mm)	Axial Cutting Depth, *a*_p_ (mm)
For evaluating the table feed’s influence on the milling process:					
12	Conventional	25, 80, 160, 200	315	1	7
For evaluating the spindle rpm’s influence on the milling process:					
12	Conventional	25	315, 630, 1250, 2000	1	7
For evaluating the radial cutting depth’s influence on the milling process:					
12	Conventional	25	630	5, 4, 3, 2, 1	7
For evaluating the axial cutting depth’s influence on the milling process:					
12	Conventional	25	315	1	12, 7, 2
For evaluating the mill diameter’s influence on the milling process:					
12, 8	Conventional	25, 80, 160, 200	315	1	7
For evaluating the cutting configuration’s influence on the milling process:					
8	Conventional, climb	25, 80, 160, 200	315	1	7

**Table 4:** Machining parameters for the deposited sample.

Machining Parameter	Table Feed, *v*_f_	Spindle Speed, *n*	Radial Depth of Cut, *a*_e_	Axial Depth of Cut, *a*_p_	Cutter Diameter, *d*	Milling Strategy
Cutting force, *F*_to_	Very high	Moderate	High	Low	Small	Complex
Surface roughness, *Ra*	Very high	Moderate	Low	Low	Very large	Very high
Vibration displacement, *x*	Very high	Moderate	Moderate	Low	Large	Very high
Temperature, *T*	High	Moderate	Very high	Low	Moderate	Moderate

## Data Availability

The original contributions presented in this study are included in the article/Supplementary Materials. Further inquiries can be directed to the corresponding authors.

## Supplementary Materials

The following supporting information can be downloaded at: https://www.mdpi.com/article/10.3390/ma19030567/s1.
