# Supplementary material for: Milling Parameters and Quality of Machined Surface of Wire Arc Additive Manufactured AISI 321 Steel
Source: Materials (Basel). 2026 Feb 2;19(3):567. doi: 10.3390/ma19030567 (PMC12898575; doi:10.3390/ma19030567)
Supplement: Supplementary file 1 [file materials-19-00567-s001.zip › materials-4093786-SI.pdf]

	Interlayer				Interlayer+Interpass				Interpass				Fusion zone			
	Measured$_1$	Measured$_2$	Measured$_3$	Average	Measured$_1$	Measured$_2$	Measured$_3$	Average	Measured$_1$	Measured$_2$	Measured$_3$	Average	Measured$_1$	Measured$_2$	Measured$_3$	Average
At the bottom region	188.55	192.76	196.97	192.76±4.21	180.60	185.75	192.40	186.25±5.92	214.41	199.51	206.43	206.78±7.46	195.61	192.43	176.81	188.28±10.06
At the center region	194.13	193.75	197.51	195.13±2.07	195.42	189.96	189.70	191.69±3.23	197.23	214.06	202.90	204.73±8.56	180.72	181.61	185.83	182.72±2.73
At the top region	206.73	197.75	194.77	199.75±6.23	187.56	194.23	196.15	192.65±4.51	198.42	209.12	205.43	204.32±5.43	185.71	182.46	181.45	183.21±2.23
At the edge region	203.18	197.45	208.02	202.63±5.29	193.41	196.25	188.75	192.80±3.79	199.87	205.19	212.45	205.84±6.31	184.54	191.25	186.35	187.38±3.47

	At the bottom region				At the center region				At the top region				At the edge region			
	Measured$_1$	Measured$_2$	Measured$_3$	Average	Measured$_1$	Measured$_2$	Measured$_3$	Average	Measured$_1$	Measured$_2$	Measured$_3$	Average	Measured$_1$	Measured$_2$	Measured$_3$	Average
Values	631.25	625.61	619.82	625.56±5.72	618.42	622.00	624.57	621.66±3.09	614.61	635.21	622.45	624.09±10.40	616.73	638.26	632.42	629.14±11.13

Cutting force

Cutter diameter, d=12mm / Conventional milling / Spindle Speed, n=315rpm / Radial Depth of Cut, a_e = 1mm / Axial Depth of Cut, a_p=7mm

Table Feed v_f, mm/min	Fx(Ph), N						Fy(Pv), N						Fz(Px), N						F_{to}, N
	Measured$_1$	Measured$_2$	Measured$_3$	Measured$_4$	Measured$_5$	Average	Measured$_1$	Measured$_2$	Measured$_3$	Measured$_4$	Measured$_5$	Average	Measured$_1$	Measured$_2$	Measured$_3$	Measured$_4$	Measured$_5$	Average	
25	-462.05	-459.63	-466.49	-463.31	-459.35	-462.17±2.93	173.21	177.51	171.22	180.61	174.75	175.46±3.68	-84.37	-82.15	-86.21	-84.35	-85.35	-84.49±1.52	501.52
80	-714.63	-714.43	-706.46	-735.21	-702.65	-714.68±12.59	303.23	295.11	288.61	324.25	304.25	303.09±13.44	-148.15	-156.69	-155.31	-156.55	-170.31	-157.40±8.028	792.09
160	-1073.21	-1097.45	-1115.43	-1068.21	-1086.14	-1088.08±19.07	347.15	362.06	357.85	377.45	337.51	356.40±15.16	-225.71	-217.81	-241.45	-232.99	-245.41	-232.67±11.28	1168.36
200	-1465.15	-1463.41	-1460.90	-1432.51	-1482.15	-1460.82±17.90	359.42	354.50	359.41	352.21	347.31	354.57±5.13	-297.68	-291.65	-288.45	-308.51	-304.21	-298.10±8.38	1532.51

Cutter diameter, d=12mm / Conventional milling / Table Feed, v_f=25mm/min / Radial Depth of Cut, a_e = 1mm / Axial Depth of Cut, a_p=7mm

Spindle Speed n, rpm	Fx(Ph), N						Fy(Pv), N						Fz(Px), N						F_{to}, N
	Measured$_1$	Measured$_2$	Measured$_3$	Measured$_4$	Measured$_5$	Average	Measured$_1$	Measured$_2$	Measured$_3$	Measured$_4$	Measured$_5$	Average	Measured$_1$	Measured$_2$	Measured$_3$	Measured$_4$	Measured$_5$	Average	
315	-462.05	-459.63	-466.49	-463.31	-459.35	-462.17±2.93	173.21	177.51	171.22	180.61	174.75	175.46±3.68	-84.37	-82.15	-86.21	-84.35	-85.35	-84.49±1.52	501.52
630	-265.31	-273.41	-274.31	-264.30	-241.61	-263.78±13.21	116.54	129.82	121.20	113.65	121.45	120.53±6.14	-67.55	-63.31	-70.65	-68.19	-69.19	-67.77±2.76	297.83
1250	-176.45	-188.46	-182.82	-191.65	-169.51	-181.78±8.98	118.41	104.34	109.28	109.43	115.42	111.37±5.56	-64.45	-70.51	-68.81	-70.31	-76.55	-70.12±4.34	224.42
2000	-132.57	-135.61	-125.43	-145.18	-126.35	-133.03±8.01	93.81	96.45	101.21	89.56	94.18	95.04±4.25	-58.41	-60.66	-60.41	-52.51	-58.12	-58.02±3.29	173.48

Cutter diameter, d=12mm / Conventional milling /Table Feed, v_f=25mm/min / Spindle Speed, n=630pm / Axial Depth of Cut, a_p=7mm

Radial Depth of Cut a_e, mm	Fx(Ph), N						Fy(Pv), N						Fz(Px), N						F_{to}, N
	Measured$_1$	Measured$_2$	Measured$_3$	Measured$_4$	Measured$_5$	Average	Measured$_1$	Measured$_2$	Measured$_3$	Measured$_4$	Measured$_5$	Average	Measured$_1$	Measured$_2$	Measured$_3$	Measured$_4$	Measured$_5$	Average	
5	-563.68	-553.19	-572.43	-572.15	-554.21	-563.13±9.31	-325.41	-337.94	-353.21	-321.54	-348.55	-337.33±13.87	-101.45	-108.17	-109.21	-113.23	-111.78	-108.77±4.56	665.39
4	-548.45	-536.51	-543.23	-560.36	-534.48	-544.60±10.40	-233.56	-236.41	-239.67	-238.32	-242.35	-238.06±3.32	-101.45	-109.45	-101.21	-105.12	-111.45	-105.73±4.62	603.69
3	-482.31	-498.06	-484.55	-512.35	-512.41	-497.94±14.50	-164.90	-154.52	-176.31	-173.21	-152.45	-164.28±10.72	-97.16	-100.35	-99.09	-102.56	-103.52	-100.53±2.57	533.89
2	-415.75	-440.21	-449.82	-423.51	-430.05	-431.87±13.46	-53.21	-59.21	-55.77	-57.23	-60.05	-57.10±2.74	-84.67	-81.44	-87.65	-87.83	-80.76	-84.47±3.33	443.74
1	-265.31	-273.41	-274.31	-264.30	-241.61	-263.78±13.21	116.54	129.82	121.20	113.65	121.45	120.53±6.14	-67.55	-63.31	-70.65	-68.19	-69.19	-67.77±2.76	297.83

Cutter diameter, d=12mm / Conventional milling /Table Feed, v_f=25mm/min / Spindle Speed, n=315pm / Radial Depth of Cut, a_e = 1mm

Axial Depth of Cut a_p, mm	Fx(Ph), N						Fy(Pv), N						Fz(Px), N						F_{to}, N
	Measured$_1$	Measured$_2$	Measured$_3$	Measured$_4$	Measured$_5$	Average	Measured$_1$	Measured$_2$	Measured$_3$	Measured$_4$	Measured$_5$	Average	Measured$_1$	Measured$_2$	Measured$_3$	Measured$_4$	Measured$_5$	Average	
2	-479.62	-493.51	-486.47	-481.25	-491.33	-486.43±6.06	159.18	155.91	152.56	164.52	162.97	159.02±4.93	-86.50	-83.15	-82.71	-89.65	-87.81	-85.96±2.99	518.93
7	-462.05	-459.63	-466.49	-463.31	-459.35	-462.17±2.93	173.21	177.51	171.22	180.61	174.75	175.46±3.68	-84.37	-82.15	-86.21	-84.35	-85.35	-84.49±1.52	501.52
12.5	-375.69	-363.45	-372.41	-393.51	-378.62	-376.73±10.96	176.55	175.55	183.52	179.02	179.15	178.75±3.08	-31.25	-35.16	-34.05	-32.22	-33.40	-33.21±1.53	418.31

Cutter diameter, d=8mm / Conventional milling / Spindle Speed, n=315rpm / Radial Depth of Cut, a_e = 1mm / Axial Depth of Cut, a_p=7mm

Table Feed v_f, mm/min	Fx(Ph), N						Fy(Pv), N						Fz(Px), N						F_{to}, N
	Measured$_1$	Measured$_2$	Measured$_3$	Measured$_4$	Measured$_5$	Average	Measured$_1$	Measured$_2$	Measured$_3$	Measured$_4$	Measured$_5$	Average	Measured$_1$	Measured$_2$	Measured$_3$	Measured$_4$	Measured$_5$	Average	
25	-564.29	-553.43	-572.81	-574.62	-556.66	-564.36±9.42	174.34	176.88	182.45	171.66	180.45	177.15±4.38	-44.69	-42.68	-41.52	-47.81	-46.25	-44.59±2.55	593.19
80	-758.41	-772.15	-766.01	-770.15	-762.55	-765.85±5.57	244.56	258.62	256.77	251.04	246.81	251.56±6.09	-103.21	-106.64	-108.79	-105.45	-110.21	-106.86±2.75	813.16
160	-1122.65	-1151.51	-1130.55	-1126.55	-1133.75	-1133.00±11.16	262.94	255.81	269.77	258.41	267.55	262.89±5.90	-192.45	-186.56	-197.51	-189.65	-178.77	-188.98±6.98	1178.35
200	-1262.33	-1269.06	-1285.43	-1255.68	-1274.78	-1269.45±11.44	273.51	279.65	274.52	276.66	275.21	275.91±2.38	-210.55	-200.88	-222.87	-230.92	-217.06	-216.45±11.49	1317.00

Cutter diameter, d=8mm / climb milling / Spindle Speed, n=315rpm / Radial Depth of Cut, a_e = 1mm / Axial Depth of Cut, a_p=7mm

Table Feed v_f, mm/min	Fx(Ph), N						Fy(Pv), N						Fz(Px), N						F_{to}, N
	Measured$_1$	Measured$_2$	Measured$_3$	Measured$_4$	Measured$_5$	Average	Measured$_1$	Measured$_2$	Measured$_3$	Measured$_4$	Measured$_5$	Average	Measured$_1$	Measured$_2$	Measured$_3$	Measured$_4$	Measured$_5$	Average	
25	-397.65	-404.07	-410.67	-405.77	-409.78	-405.58±5.21	575.65	580.63	582.45	576.38	588.41	580.70±5.16	-76.43	-78.35	-79.28	-73.21	-71.55	-75.76±3.30	712.35
80	-667.74	-653.57	-672.15	-676.31	-660.89	-666.13±9.05	933.42	940.72	943.31	927.51	931.45	935.28±6.56	-158.71	-162.32	-161.88	-156.23	-170.19	-161.86±5.27	1159.60
160	-341.21	-349.45	-345.78	-353.25	-343.41	-346.62±4.80	590.74	583.51	597.62	595.58	603.51	594.19±7.52	-68.91	-69.28	-75.43	-76.25	-71.24	-72.22±3.43	691.68
200	-143.21	-151.28	-140.77	-149.12	-147.30	-146.33±4.29	351.29	357.87	357.24	360.78	367.21	358.87±5.79	-17.40	-16.29	-17.94	-15.91	-18.55	-17.21±1.10	387.94

Temperature *T* + Vibration displacement *x* + Surface roughness *Ra*

Cutter diameter, d=12mm / Conventional milling / Spindle Speed, n=315rpm / Radial Depth of Cut, a_e = 1mm / Axial Depth of Cut, a_p=7mm

Table Feed v_f, mm/min	*T*						*x*						*Ra*					
	Measured$_1$	Measured$_2$	Measured$_3$	Measured$_4$	Measured$_5$	Average	Measured$_1$	Measured$_2$	Measured$_3$	Measured$_4$	Measured$_5$	Average	Measured$_1$	Measured$_2$	Measured$_3$	Measured$_4$	Measured$_5$	Average
25	25	26	24	26	24	25±1.00	25.47	25.23	25.95	24.25	26.17	25.41±0.75	0.607	0.612	0.603	0.612	0.601	0.607±0.005
80	31	33	32	32	33	32.2±0.83	39.15	40.98	40.30	41.02	40.55	40.40±0.75	1.107	1.113	1.123	1.110	1.133	1.117±0.011
160	42	39	39	40	40	40.0±1.22	53.81	56.21	55.45	54.15	54.91	54.90±0.97	1.499	1.521	1.533	1.584	1.525	1.532±0.031

200	47	48	48	46	50	47.8±1.48	89.77	91.024	92.05	90.88	91.25	90.99±0.82	2.215	2.301	2.389	2.443	2.342	2.338±0.086
Cutter diameter, d=12mm / Conventional milling / Table Feed, v_f=25mm/min / Radial Depth of Cut, a_e = 1mm / Axial Depth of Cut, a_p=7mm																		
Spindle Speed n, rpm	*T*						*x*						*Ra*					
	Measured$_1$	Measured$_2$	Measured$_3$	Measured$_4$	Measured$_5$	Average	Measured$_1$	Measured$_2$	Measured$_3$	Measured$_4$	Measured$_5$	Average	Measured$_1$	Measured$_2$	Measured$_3$	Measured$_4$	Measured$_5$	Average
315	25	26	24	26	24	25±1.00	25.47	25.23	25.95	24.25	26.17	25.41±0.75	0.607	0.612	0.603	0.612	0.601	0.607±0.005
630	29	28	30	31	31	29.8±1.30	41.52	41.16	40.36	38.25	40.36	40.33±1.26	0.591	0.583	0.594	0.602	0.608	0.595±0.009
1250	32	33	35	32	34	33.2±1.30	49.21	49.52	50.65	50.73	47.81	49.58±1.19	0.577	0.588	0.578	0.602	0.587	0.586±0.010
2000	37	33	36	35	35	35.2±1.48	50.35	52.98	54.55	51.17	52.34	52.27±1.62	0.734	0.772	0.751	0.753	0.763	0.754±0.014
Cutter diameter, d=12mm / Conventional milling /Table Feed, v_f=25mm/min / Spindle Speed, n=630pm / Axial Depth of Cut, a_p=7mm																		
Radial Depth of Cut a_e, mm	*T*						*x*						*Ra*					
	Measured$_1$	Measured$_2$	Measured$_3$	Measured$_4$	Measured$_5$	Average	Measured$_1$	Measured$_2$	Measured$_3$	Measured$_4$	Measured$_5$	Average	Measured$_1$	Measured$_2$	Measured$_3$	Measured$_4$	Measured$_5$	Average
5	58	56	57	60	60	58.2±1.78	67.32	68.16	69.45	70.18	65.45	68.11±1.85	0.745	0.792	0.775	0.791	0.771	0.774±0.019
4	45	47	48	51	50	48.2±2.38	57.23	55.45	58.19	56.23	60.18	57.45±1.83	0.658	0.705	0.713	0.694	0.685	0.691±0.021
3	39	40	42	39	40	40.0±1.22	40.15	45.32	46.21	42.99	40.77	43.08±2.68	0.653	0.625	0.636	0.616	0.645	0.635±0.014
2	35	34	37	35	33	34.8±1.48	36.74	37.82	39.21	38.15	36.98	37.78±0.98	0.575	0.563	0.612	0.594	0.634	0.595±0.028
1	29	28	30	31	31	29.8±1.30	41.52	41.16	40.36	38.25	40.36	40.33±1.26	0.591	0.583	0.594	0.602	0.608	0.595±0.009
Cutter diameter, d=12mm / Conventional milling /Table Feed, v_f=25mm/min / Spindle Speed, n=315pm / Radial Depth of Cut, a_e = 1mm																		
Axial Depth of Cut a_p, mm	*T*						*x*						*Ra*					
	Measured$_1$	Measured$_2$	Measured$_3$	Measured$_4$	Measured$_5$	Average	Measured$_1$	Measured$_2$	Measured$_3$	Measured$_4$	Measured$_5$	Average	Measured$_1$	Measured$_2$	Measured$_3$	Measured$_4$	Measured$_5$	Average
2	31	30	27	29	33	30±2.23	24.15	24.50	24.37	23.99	25.11	24.42±0.43	0.594	0.562	0.591	0.602	0.618	0.593±0.020
7	25	26	24	26	24	25±1.00	25.47	25.23	25.95	24.25	26.17	25.41±0.75	0.607	0.612	0.603	0.612	0.601	0.607±0.005
12.5	29	25	25	27	30	27.2±2.28	26.87	27.57	27.41	26.78	27.18	27.16±0.33	0.605	0.652	0.621	0.621	0.610	0.621±0.018
Cutter diameter, d=8mm / Conventional milling / Spindle Speed, n=315rpm / Radial Depth of Cut, a_e = 1mm / Axial Depth of Cut, a_p=7mm																		
Table Feed v_f, mm/min	*T*						*x*						*Ra*					
	Measured$_1$	Measured$_2$	Measured$_3$	Measured$_4$	Measured$_5$	Average	Measured$_1$	Measured$_2$	Measured$_3$	Measured$_4$	Measured$_5$	Average	Measured$_1$	Measured$_2$	Measured$_3$	Measured$_4$	Measured$_5$	Average
25	24	25	26	26	23	24.8±1.30	32.05	34.52	33.12	35.21	38.45	34.67±2.44	1.080	1.076	0.978	1.135	1.127	1.079±0.062
80	29	28	25	31	32	29±2.73	40.54	45.25	47.31	42.91	39.85	43.17±3.14	2.315	1.978	2.267	2.456	2.317	2.266±0.176
160	31	32	33	34	36	33.2±1.92	42.25	46.34	47.48	50.01	44.85	46.18±2.89	3.272	3.006	3.773	3.782	3.467	3.460±0.332
200	33	36	33	35	34	34.2±1.30	45.21	49.63	44.25	50.11	47.13	47.26±2.59	3.758	4.080	4.215	4.144	4.155	4.070±0.181
Cutter diameter, d=8mm / climb milling / Spindle Speed, n=315rpm / Radial Depth of Cut, a_e = 1mm / Axial Depth of Cut, a_p=7mm																		
Table Feed v_f, mm/min	*T*						*x*						*Ra*					
	Measured$_1$	Measured$_2$	Measured$_3$	Measured$_4$	Measured$_5$	Average	Measured$_1$	Measured$_2$	Measured$_3$	Measured$_4$	Measured$_5$	Average	Measured$_1$	Measured$_2$	Measured$_3$	Measured$_4$	Measured$_5$	Average
25	25	27	24	28	21	25±2.73	56.28	51.45	58.28	61.01	53.47	56.09±3.78	0.958	0.915	1.023	0.944	0.901	0.948±0.047
80	35	39	36	41	43	38.8±3.34	61.02	63.05	59.75	67.01	65.78	63.32±3.07	1.016	1.125	1.118	1.214	1.154	1.125±0.071
160	35	34	36	37	37	35.8±1.30	62.41	54.98	59.11	61.41	57.21	59.02±3.03	1.176	1.227	1.316	1.402	1.123	1.248±0.111
200	31	31	33	32	30	31.4±1.14	52.20	53.15	59.01	55.45	55.20	55.00±2.62	1.175	1.432	1.502	1.265	1.054	1.285±0.183
